# Bermuda as an Evolutionary Life Raft for an Ancient Lineage of Endangered Lizards

**DOI:** 10.1371/journal.pone.0011375

**Published:** 2010-06-30

**Authors:** Matthew C. Brandley, Yuezhao Wang, Xianguang Guo, Adrián Nieto Montes de Oca, Manuel Fería Ortíz, Tsutomu Hikida, Hidetoshi Ota

**Affiliations:** 1 Museum of Vertebrate Zoology and Department of Integrative Biology, University of California, Berkeley, California, United States of America; 2 Chengdu Institute of Biology, Chinese Academy of Sciences, Chengdu, China; 3 Departamento de Biología Evolutiva, Facultad de Ciencias, Universidad Nacional Autónoma de México, México, Distrito Federal, México; 4 Department of Zoology, Graduate School of Science, Kyoto University, Kyoto, Japan; 5 Institute of Natural and Environmental Sciences and Museum of Nature and Human Activities, University of Hyogo, Hyogo, Japan; University of Zurich, Switzerland

## Abstract

Oceanic islands are well known for harboring diverse species assemblages and are frequently the basis of research on adaptive radiation and neoendemism. However, a commonly overlooked role of some islands is their function in preserving ancient lineages that have become extinct everywhere else (paleoendemism). The island archipelago of Bermuda is home to a single species of extant terrestrial vertebrate, the endemic skink *Plestiodon* (formerly *Eumeces*) *longirostris*. The presence of this species is surprising because Bermuda is an isolated, relatively young oceanic island approximately 1000 km from the eastern United States. Here, we apply Bayesian phylogenetic analyses using a relaxed molecular clock to demonstrate that the island of Bermuda, although no older than two million years, is home to the only extant representative of one of the earliest mainland North American *Plestiodon* lineages, which diverged from its closest living relatives 11.5 to 19.8 million years ago. This implies that, within a short geological time frame, mainland North American ancestors of *P. longirostris* colonized the recently emergent Bermuda and the entire lineage subsequently vanished from the mainland. Thus, our analyses reveal that Bermuda is an example of a “life raft” preserving millions of years of unique evolutionary history, now at the brink of extinction. Threats such as habitat destruction, littering, and non-native species have severely reduced the population size of this highly endangered lizard.

## Introduction

*“…it appears that the true history of the colonization of the land, now Bermuda, is lost forever in oblivion”* [1]

Studies of island biodiversity have focused largely on adaptive radiations associated with neoendemism (i.e., “cradles” of diversity) [2]–[4]. There are myriad factors that promote spectacular biodiversity on islands, but the factors that contribute to neoendemism ultimately derive from the fact that the islands formed *de novo* with no connection to a larger landmass and thus have abundant “empty” ecological niche space. However, the isolation that defines islands can also preserve genetic diversity of relict lineages, a pattern known as paleoendemism [3], [5]. The most prominent example of this phenomenon among vertebrates is the tuatara (*Sphenodon*) that represents a clade of reptiles once widespread, but now restricted to two remaining species found only on the offshore islands of New Zealand. Moreover, given numerous threats such as climate change, introduced species, and habitat destruction, insular fauna are subject to increased risk of extinction [6]. This vulnerability is of particular concern for paleoendemics, as these taxa represent a disproportionately high amount of phylogenetic diversity [7]–[9].

**Figure 1:**
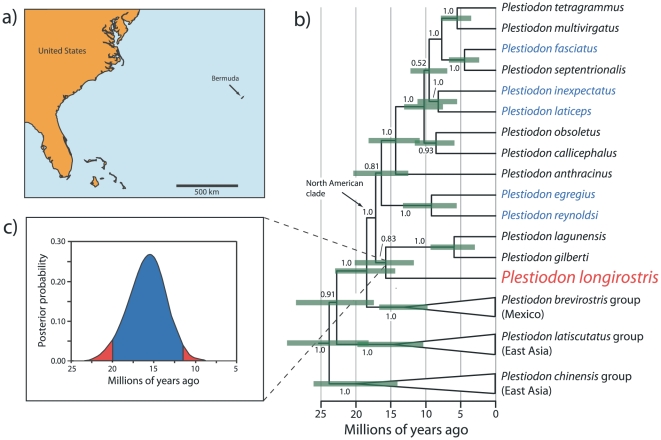
The location of Bermuda, phylogeny of *Plestodon*, and molecular estimates of divergence times. a., Map showing the location of Bermuda relative to North America. b., Phylogeny of the genus *Plestiodon* (outgroups not shown; see Brandley et al, 2010). Branch lengths are in units of time and represent the means of the posterior distribution. Numbers above or below the nodes indicate posterior probabilities. Triangles indicate groups for which multiple species were sampled, but are not shown. Taxa in blue are those that inhabit eastern North America. Green bars indicate the 95% credible interval for estimated divergence dates for that node. c., Posterior probability distribution of the age of the divergence between *Plestiodon longirostris* and its sister lineage. Areas shaded in red are values that exceed the 95% credible interval.

The islands of Bermuda (32.33°N, 64.75°W) are an isolated, 54 km^2^ archipelago (referred to as “island”, hereafter) approximately 1000 km east of the United States (Fig. 1a). The island is currently home to a single endemic terrestrial vertebrate, the scincid lizard *Plestiodon longirostris* (formerly *Eumeces longirostris* [10], [11]), although several other terrestrial vertebrates, including a tortoise (*Hesperotestudo*) and multiple species of birds, inhabited the island until from the Middle to Late Pleistocene [12]–[19]. *Plestiodon longriostris* is currently considered critically endangered [20] and faces continuing threats of extinction through human-caused habitat loss, competition with and predation from introduced species, and entrapment in discarded bottles [21]. Once abundant [22], [23], the species' range has dwindled to several small sub-populations, the largest of which occurs on Southampton Island and contains only an estimated 400 individuals [21]. Preliminary microsatellite analysis indicates that the current genetic diversity of *P. longirostris* is low [24].

Pre-plate tectonics biogeographic hypotheses assumed that *P. longirostris* was a relict of an ancient lineage that became isolated on Bermuda after the closing of a land bridge [1]. This “relict” hypothesis was also influenced by the species' unique morphological characteristics (including an elongated snout). However, recent geologic studies have demonstrated that, although part of an ancient, volcanic sea-mount, most of the exposed, terrestrially habitable portion of Bermuda has never been connected to a larger landmass and instead consists of limestone deposited in the Pleistocene [25]–[28], the maximum age of which is no more than two million years [29]. Moreover, all known Bermudan vertebrate fossils are limited to sediments from the latter half of the Pleistocene [13]–[19]. A more reasonable alternative hypothesis is that the species is quite young, at most as old as the maximum age of Bermuda (1–2 million years), and is a descendent of one of the several species of *Plestiodon* inhabiting the eastern United States that dispersed over water to Bermuda following the emergence of the island. Another reptile (indeed, the only other potentially native reptile), the diamondback terrapin (*Malaclemys terrapin*), is a likely very recent immigrant that descended from populations of the same species that currently inhabit the eastern United States [30], [31].

To evaluate the evolutionary history of this unique skink, and specifically to test the “relict” and “recent immigrant” hypotheses, we conducted Bayesian relaxed molecular clock divergence dating analyses of an extensive, multi-locus DNA data set including all *Plestiodon* species from the eastern United States and the Bermudan *P. longirostris*. We demonstrate that the oceanic island of Bermuda, despite its young age (no more than two million years) and history of extreme changes in available habitat, harbors the last representative of one of the earliest North American lineages of *Plestiodon* that diverged ∼16 million years ago.

## Results and Discussion

The results of the phylogenetic analysis (pruned to show relevant taxa only; Fig. 1b) show that the lineage that includes modern *P. longirostris* does not descend from any of the extant *Plestiodon* lineages that inhabit eastern North America. In fact, Bayesian divergence dating analyses using a relaxed molecular clock indicate that it is one of the earliest diverging lineages of the entire North American clade (Figs. 1b,c). Furthermore, this divergence occurred ∼16 million years ago (Ma) (95% credible interval = 11.5 to 19.8 Ma), well before any modern species, and the lineage contains no extant representative other than *P. longirostris*. We are therefore left with the remarkable conclusion that a two million-year-old island contains the sole survivor of an ancient lineage that predates the existence of Bermuda by well over 10 million years.

Although no fossils of the *P. longirostris* lineage are known from mainland North America [1], the phylogenetic and divergence date data clearly indicate that ancestors once existed on the mainland. However, *P. longirostris* is present in Bermudan fossil beds dated to approximately 400,000 years ago. Given the young age of Bermuda, these results support the hypothesis that within the past 400,000 to two million years, individuals from the North American mainland *P. longirostris* lineage dispersed to a presumably recently emerged Bermuda 1000 km offshore, yet subsequently became extinct on the mainland. Although we can only speculate how these colonizing individuals dispersed over water, we note that both hurricanes and ocean currents are known to transport living lizards and debris to and from islands [32]–[33], and that the powerful Gulf Stream ocean current runs along eastern North America to the mid-Atlantic Ocean [see 31].

Thus, despite its young age, the island preserves the last representative of one of the oldest lineages of mainland North American *Plestiodon* – it is essentially an evolutionary “life raft”. This “life raft” role is remarkable considering that extreme fluctuations in sea level during the Pleistocene have intermittently decreased the available terrestrial habitat on Bermuda by orders of magnitude [18]. This extreme contraction in habitat was the likely cause of the extinction of several endemic birds [13]–[19]. Bermuda's only other native reptile, *Malaclemys terrapin*, only colonized the island in the past 3000 to 400 years from populations that currently inhabit the Eastern United States [30].

These results are extremely unlikely to be the result of error in divergence date estimates or phylogenetic uncertainty. One advantage of Bayesian methods of divergence dating is their ability to incorporate error in both the calibration age constraint and phylogeny, and to infer posterior probability distributions of estimated ages. The 95% credible interval of the date of divergence between *P. longirostris* and other North American *Plestiodon* species ranges from 11.5 to 19.8 Ma and excludes the earliest date that Bermuda may have emerged (2 Ma). Although the relationship between *P. longirostris* and its sister lineage is not well supported (posterior probability<0.95), this species is nonetheless excluded from any other younger clades with strong statistical support (Fig. 1b). In other words, the lineage cannot be any younger than the other major clades that all predate the emergence of Bermuda. Furthermore, if this lack of resolution represents a rapid radiation at the base of the North American *Plestiodon* phylogeny, then the age of the *P. longirostris* lineage is even older (∼13–21 Ma; Fig. 1b). Finally, extensive analyses of this data set have demonstrated that the use of models that account for rate heterogeneity among subsets of the DNA dramatically improve divergence date estimates and help mitigate potential problems inherent in using distantly related age calibrations [34].

We also note that the history of *P. longirostris* somewhat parallels that of the extinct Bermudan turtle, *Hesperotestudo bermudae*. Fossil evidence indicates this species became extinct on Bermuda 300,000 years ago, yet was the last representative of a genus of tortoise that inhabited North America from the Oligocene to Pleistocene [13], [18]. With the caveat that more recent fossils of North American *Hesperotestudo* may yet to be discovered, these data currently suggest that Bermuda also served as an evolutionary life raft for this genus after extinction on the mainland.

Although we certainly do not discount the profoundly important role of islands in generating biodiversity, our results highlight the frequently overlooked role of islands in preserving diversity (acting as evolutionary “museums”). This role is of particular importance given that preservation of phylogenetic diversity has been an increasingly important goal of conservation biology as the extinction of “old” species would result in a greater loss of genetic diversity than that of a “young” species with close phylogenetic relatives [7]–[9]. Therefore, because the Bermuda skink, *Plestiodon longirostris*, represents the sole representative of one of the earliest diverging lineages among North American *Plestiodon*, efforts to preserve this species are also preserving ∼12 to 20 millions of years of unique evolutionary history at the risk of extinction.

## Materials and Methods

### Taxon and character sampling

DNA for 62 individuals representing 37 of ∼43 recognized species of *Plestiodon* and 25 outgroups was isolated from tissue using Qiagen DNeasy™ columns (see [34] for detailed specimen information and methods). We amplified BDNF, MKL, mtDNA [ND1+tRNAs], PRLR, PTGER4, R35, RAG1, and SNCAIP loci using standard PCR techniques (Genbank numbers upon acceptance). In few cases, we were unable to obtain reliable sequences for some species; in this case, another species from the same family was used. PCR products were cleaned using ExoSap-IT (USB Corp.). Purified templates were dye-labeled using BigDye™ (ABI) and sequenced on an ABI 3077™ automated DNA sequencer. Nucleotide sequences were examined and aligned by eye. This process was relatively straightforward for the protein-coding genes (BDNF, MKL, mtDNA ND1, PRLR, PTGER4, R35, RAG1, and SNCAIP) due to their codon reading frames. MtDNA tRNAs were aligned according to their secondary structure, and regions in which homology was uncertain due to multiple insertions and deletions were excluded from subsequent analysis. The size of the final data set for phylogenetic analysis was 7667 bp.

### Phylogenetic analyses

Brandley et al. [34] demonstrated that accommodating different rates of evolution among subsets of DNA data may improve divergence time estimation, especially when different subsets of the data evolve at different rates. We therefore partitioned the data *a priori* by locus and codon position (and a single partition for the tRNAs) for a total of 28 partitions. For each partition, we determined the appropriate model of nucleotide substitution using the Bayesian information criterion (BIC) [35].

All phylogenetic analyses of the combined data set were conducted using BEAST v1.4.8 [36] assuming an uncorrelated lognormal relaxed molecular clock [37]. A total of seven analyses were performed. Each analysis used a coalescent starting tree and was run for 10^8^ generations, sampled every 10,000^th^ generation. We used the program's default prior distributions with the exception of GTR substitution rates in which we used a uniform (0,100) distribution, and the date distributions of the most recent common ancestor of the three clades used for calibration (see below). To determine convergence, we constructed cumulative posterior probability plots for each clade using the *cumulative* and *compare* function in AWTY [38]. Posterior probabilities≥0.95 are considered statistically significant clade support [39].

Because the *Plestiodon* fossil record, and the record of fossil skinks in general, are extremely poor, we used three fossil calibration age prior distributions from non-scincid fossil taxa whose phylogenetic placement in the squamate tree was recently inferred [40]. The age of crown Episquamata (represented here as *Anniella, Aspidoscelis*, *Basiliscus*, and *Bipes*) was calibrated using the age of the earliest stem “anguimorph” fossils, *Becklesius, Dorsetisaurus*, *Paramacellodus*, and *Pseudosaurilius* [34], [40]. We chose a lognormal distribution so that the earliest possible sampled age corresponds to 148 Ma and the older 97.5% credible interval (CI) encompasses the earliest age of crown Squamata (180 Ma: standard deviation = 1.769; [40], [41]). The age of the divergence between Amphisbaenia (*Bipes biporus*) and Teiidae (*Aspidoscelis*) was calibrated using the age (Albian - Cenomanian boundary) of the earliest teiioid (Polyglyphanodontidae) fossils, (e.g., *Bicuspidon* [40], [42]). We chose a lognormal distribution so that the earliest possible sampled age corresponds to 96 Ma and the older 97.5% credible interval (CI) encompasses the earliest age of crown Episquamata (148 Ma; standard deviation = 2.016). The age of Scinciformata (represented here by skinks, Gerrhosauridae, and Xantusiidae) was calibrated using the age (Berriasian) of the fossil *Sakurasaurus* [40], [43]. We chose a lognormal distribution so that the earliest possible sampled age corresponds to 138 Ma and the older 97.5% credible interval (CI) encompasses the earliest age of the root (151 Ma; standard deviation = 1.309). We therefore enforced the monophyly of these clades in accordance with recent phylogenetic analyses that have inferred these relationships [44], [45]. The full phylogeny, including all outgroups, is provided in Fig. S1.

## Supporting Information

Figure S1 Full phylogeny including fossil calibrations (in red). Boxes indicate 95%CIs of node ages.
(1.48 MB EPS)
